# Network meta-analysis on 4 types of traditional Chinese medicine injections in the treatment of senile severe pneumonia

**DOI:** 10.1097/MD.0000000000041060

**Published:** 2024-12-27

**Authors:** Yangli Tu, Changbing Wu, Liang Chen, Yinzhi Cen, Jie Liu

**Affiliations:** a Guiyang Fourth People’s Hospital, Guiyan, Guizhou Province, China; b College of Pharmacy, Guizhou Medical University, Guiyan, Guizhou Province, China.

**Keywords:** network meta-analysis, Reduning injection, senile severe pneumonia, Tanreqing injection, Xiyanping injection, Xuebijing injection

## Abstract

**Background:**

As the global population ages, senile severe pneumonia has become a prominent health challenge. Conventional drug treatments have limitations. Meanwhile, 4 types of traditional Chinese medicine injections have shown potential. Thus, it’s necessary to use network meta-analysis to comprehensively evaluate their combined effect with conventional drugs on this disease.

**Methods:**

China National Knowledge Infrastructure, VIP shop Database, WanFang database, PubMed, and Cochrane Library were included in randomized controlled studies on traditional Chinese medicine injections for the treatment of senile severe pneumonia from the establishment of the library to January 2023. The literature was assessed for risk of bias using the Review Manager 5.3 software. Study outcome indicators were statistically analyzed using Stata 14.0 and Review Manager 5.3 software.

**Results:**

Thirty-three studies were included with a total of 2598 patients. The main outcome indicator was the clinical effective rate. Secondary outcome indicators were white blood cell count, procalcitonin, and C-reactive protein. The results of network meta-analysis showed that clinical efficacy of Reduning injection combined with conventional drug had the better effect. Xiyanping injection combined with conventional drug could better reduce white blood cell and C-reactive protein indicators. Xuebijing injection combined with conventional drug had better ability to reduce procalcitonin.

**Conclusion:**

Reduning injection, Xiyanping injection, Tanreqing injection, and Xuebijing injection combined with conventional drugs in the treatment of senile severe pneumonia can improve the clinical effect.

## 1. Introduction

Pneumonia is an inflammation that occurs primarily in the interstitium, alveoli, and terminal airways of the lungs.^[1]^ If pneumonia is left untreated, it can easily develop into a serious pneumonia.^[2]^ However, the elderly have a weaker immune system.^[3]^ Elderly people infected with pneumonia are prone to develop severe pneumonia. Previous studies have reported that the incidence of senile severe pneumonia was about 8%, while the mortality rate was 50% to 70%.^[4]^ Senile severe pneumonia is also relatively difficult to treat clinically. Anti-infection, mechanical ventilation, and systemic glucocorticoids are often used to treat severe pneumonia in the elderly.^[5,6]^ Previous researches have reported conventional drugs combined with traditional Chinese medicine injections have better clinical efficacy in the treatment of senile severe pneumonia.^[7]^ Problems such as dysphagia, gastrointestinal reactions, and avoiding the first pass elimination were solved by Chinese medicine injections.^[8]^ However, there is a lack of direct comparisons of clinical efficacy among herbal injections, and the problem of how to choose the best treatment plan is faced in clinical practice. Therefore, this study conducted a network meta-analysis of a randomized controlled to evaluation of the clinical efficacy of Xuebijing Injection, Tanreqing Injection, Xiyanping Injection, and Reduning Injection combination with conventional drugs in the treatment of elderly patients with severe pneumonia. This can provide evidence-based basis for clinical drug use.

## 2. Materials and methods

### 2.1. Inclusion criteria

#### 2.1.1. Study population

The study population was patients diagnosed with severe pneumonia according to relevant guidelines. Patients with severe pneumonia should be older than 65 years of age. Nationality, sex, race, and disease duration of patients with severe pneumonia were not limited.

#### 2.1.2. Interventions

The control group was treated with conventional drugs such as linezolid, meropenem, piperacillin sodium, tazobactam sodium, and linezolid. Expectorant, oxygen inhalation, fluid replacement, nutritional support, and mechanical ventilation. Treatment of conventional was treated with traditional Chinese medicine injection such as Xuebijing injection, Xiyanping injection, Tanreqing injection, Reduning injection on the basis of conventional drugs.

#### 2.1.3. Study type

The types of articles included in the study were randomized controlled trials studies. The language types of retrieved articles were Chinese and English.

#### 2.1.4. Outcome measures observed

The principal outcome measure was the clinical effective rate, which could be appraised through 3 levels: markedly effective, effective, and ineffective. Markedly effective implies that the symptoms and physical signs of the patients vanished, and the lung shadow had disappeared as demonstrated by a chest X-ray examination. Effective indicates that the symptoms of fever, cough, and dyspnea in the patients were ameliorated to a certain extent, yet not all disappeared, and the lung shadow contracted. Ineffective denotes that the signs and symptoms of the patients persisted or there was deterioration. The clinical effective rate was calculated as follows: (the number of markedly effective cases + the number of effective cases)/total sample size × 100%. Secondary outcome measures was C-reactive protein (CRP), white blood cell (WBC), and procalcitonin (PCT).

### 2.2. Exclusion criteria

Network Pharmacology Research; In vitro experiment; Review; Systematic review and repeated literatures; incomplete study content; non-randomized trial, etc.

### 2.3. Search strategy

China National Knowledge Infrastructure, VIP shop Database, WanFang database, PubMed, and Cochrane Library were included in randomized controlled studies on traditional Chinese medicine injections for the treatment of senile severe pneumonia from the establishment of the library to January 2023. The search for literature was performed by combining subject headings with free words and adjusted according to the characteristics of each database. The search terms included senile severe pneumonia, Xuebijing injection, Reduning injection, Tanreqing injection, Xiyanping injection, traditional Chinese medicine injection, etc, and the search terms was not limited by the type of publication.

### 2.4. Literature screening and data extraction

Literature was independently screened by 2 researchers based on inclusion and exclusion criteria. researchers extracted studies that met the inclusion criteria by reading the full text and based on the Cochrane Evaluation Handbook’s quality assessment criteria. studies were extracted information about the year, authors, interventions, and outcome indicators by Excel software.

### 2.5. Evaluation of risk of bias of included studies

literatures were assessed for risk of bias by 2 researchers independently using Review Manager 5.3 software. Then cross-check. If there is any discrepancy, ask the third investigator to assist in the judgment.

### 2.6. Statistical analysis

Study outcome indicators were statistically analyzed using Stata 14.0 and Review Manager 5.3 software. Measurement data were described with mean difference (MD) as the effect analysis statistic and its 95% confidence interval (95% CI). Enumeration data were described with odds ratio (OR) and its 95% CI. Network meta-analysis was performed using Network Group command for data preprocessing. Network evidence maps and “correction-comparison” funnel diagram were drawn for each outcome indicator. The different interventions were compared in pairs. The clinical efficacy was ranked according to area under the cumulative ranking curve surface under the cumulative ranking curve (SUCRA). Inconsistencies were checked when a closing ring was formed.

## 3. Results

### 3.1. Literature search process and results

**Figure 1.**
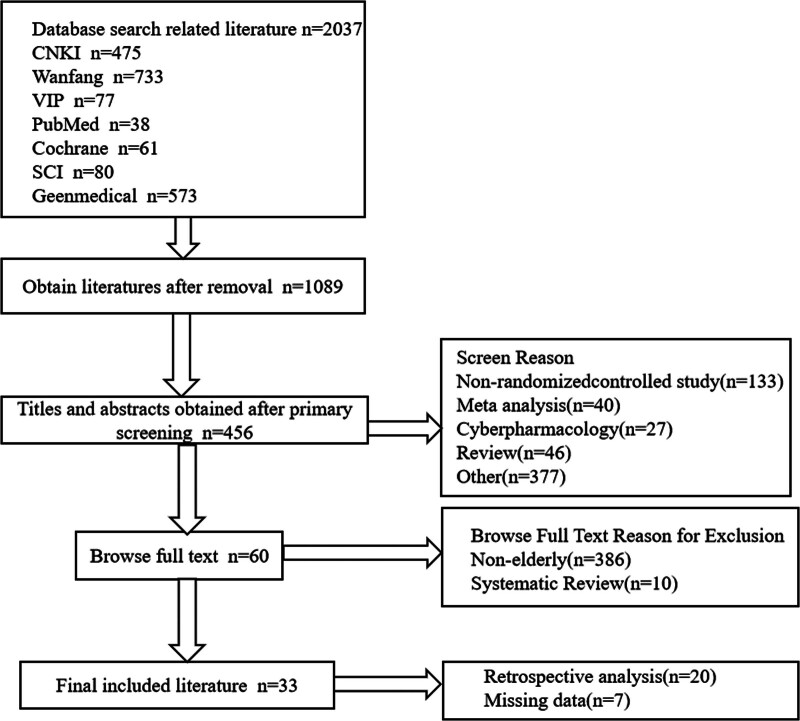
Literature screening procedure and result.

A total of 2037 relevant literatures were initially examined. Duplicate literatures were excluded by Endnote software. One thousand five hundred eighty-one literatures were excluded by reading the title and abstract according to the inclusion criteria and exclusion criteria. Three hundred ninety-six literatures were excluded by reading the full text. Finally, 33 literatures^[9–41]^were included in the meta-analysis. The literature screening process and results are shown in Figure 1.

### 3.2. Basic characteristics of included studies

There were a total of 2598 patients, including 1287 cases in the control group and 1311 cases in the experimental group. Four types of traditional Chinese medicine injections were involved, and all were two-arm experiments. There were 18 studies^[9–26]^ related to Xuebijing injection combined with conventional drug treatment. Nine studies^[27–35]^ pertained to Tanreqing injection combined with conventional drug treatment. Four studies^[36–39]^ were associated with Xiyanping injection combined with conventional drug treatment. Two studies^[40,41]^ involved Reduning injection combined with conventional drug treatment. Among them, 31 studies^[9–18,20–23,25–41]^ reported the clinical effective rate. Nineteen studies^[9–11,14–18,20,23,24,27,28,32,34,36,37,41]^ reported CRP. Sixteen studies^[10,16–20,22,24,25,27,28,34,36,37,41]^ reported WBC count. Seven studies^[10,12,16,20,23,27,28,32]^ reported PCT. There was no statistically significant difference in age, gender, and other general conditions among the patients in all studies. The basic characteristics of the included literatures are presented in Table 1.

**Table 1:** Basic characteristics of included literature.

literature resources	Sample size/cases		Age/year		Interventions		Treatment course (day)	Evaluation indicators
	T	C	T	C	T	C		
Xiao Qing et al^[9]^	48	48	69. 13 ± 5. 24	69.22 ± 4. 57	XBJ + CG	CG	14	①②
Xiao Hongwen et al^[10]^	41	41	72. 31 ± 4. 35	71. 85 ± 3. 87	XBJ + CG	CG	7	①②③④
Xu Jingjing et al^[11]^	33	33	72.42 ± 1.50	71.23 ± 1.23	XBJ + CG	CG	14	①②
Huiwen et al^[12]^	16	15	70.5 ± 9.5	69.0 ± 8.6	XBJ + CG	CG	7	①④
Li Xiaojuan et al^[13]^	53	48	70.85 ± 6.33	70.42 ± 7.95	XBJ + CG	CG	14	①
Wei Ho et al^[14]^	41	31	69.48 ± 5.21	68.76 ± 4.52	XBJ + CG	CG	14	①②
Guo Limin^[15]^	40	40	72.2 ± 6.7	72.0 ± 6.6	XBJ + CG	CG	7	①②
Chen Jinzhong^[16]^	43	43	69. 78 ± 5. 76	68. 23 ± 4. 92	XBJ + CG	CG	14	①②③④
Zhuang Le^[17]^	12	12	–		XBJ + CG	CG	7	①②③
Zhu Yajing^[18]^	50	50	71.75 ± 7.15		XBJ + CG	CG	14	①②③
Zhang Xi et a1^[19]^	62	62	67.9 ± 12.2		XBJ + CG	CG	7	③
Xiao Yang^[20]^	41	41	68.51 ± 6.21	69.29 ± 6.29	XBJ + CG	CG	7	①②③④
Attorney^[21]^	45	45	72. 7 ± 7. 0		XBJ + CG	CG	7	①
Liu Jianlin^[22]^	32	32	76.2 ± 8.5	78.5 ± 7.2	XBJ + CG	CG	14	①③
Xiao Huiyun^[23]^	60	60	–		XBJ + CCG	CG	14	①②④
Zhigang Wang^[24]^	16	16	68.7		XBJ + CG	CG	14	②③
Ming Yue et al^[25]^	56	52	72.56 ± 8.95	71.2 ± 38.92	XBJ + CG	CG	7	①③
Liu Hui^[26]^	43	42	68.23 ± 2.95	67.12 ± 3.02	XBJ + CG	CG	-	①
Zhang Hongyan^[27]^	25	25	70.53 ± 1.04	70.55 ± 1.01	TRQ + CG	CG	14	①②③④
Liu Xiaoling et al^[28]^	46	46	70.25 ± 3.33	70.16 ± 3.28	TRQ + CG	CG	14	①②③④
Xu Rugui^[29]^	55	55	71.85 ± 5.72	72.34 ± 5.66	TRQ + CG	CG	3	①
Chen Changming^[30]^	44	44	69.5土2.5	70.5 ± 2.5	TRQ + CG	CG	–	①
Li Gang et al^[31]^	23	23	65.57 ± 1.36	63.66 ± 1.45	TRQ + CG	CG	–	①
Zhou Jing et al^[32]^	38	38	71.15 ± 5.38	69.32 ± 5.16	TRQ + CG	CG	15	①②④
Gu Qin^[33]^	50	50	76.5 ± 5.6		TRQ + CG	CG	5	①
Zhang Ping et al^[34]^	24	24	65.11 ± 4.36		TRQ + CG	CG	7	①②③
Wu Xiaodong^[35]^	30	30	78. 62 ± 13. 48		TRQ + CG	CG	14	①
Lai Daokin et al^[36]^	50	50	75.3 ± 12.4	75.2 ± 12.5	XYP + CG	CG	10	①②③
Liu Rong et al^[37]^	41	41	72.69 ± 9.30	73.25 ± 9.63	XYP + CG	CG	14	①②③
Hou Yuwei^[38]^	31	31	69.36 ± 3.26	69.35 ± 2.33	XYP + CG	CG	14	①
Lili Zhang et al^[39]^	33	30	74.82 ± 12.02	73.82 ± 11.97	XYP + CG	CG	14	①
Ding Feng^[40]^	50	50	71.37 ± 5.71	71.54 ± 5.69	RDN + CG	CG	10	①
Zhou Jing^[41]^	39	39	73.54 ± 12.09	72.68 ± 11.26	RDN + CG	CG	14	①②③

T = test. C = control. – = not reported. ① Clinical effective rate, ② C-reactive protein, ③ white blood cell count, ④ procalcitonin.

CG = Routine treatment, RDN = Reduning injection, TRQ = Tanreqing injection, XBJ = Xuebijing injection, XYP = Xiyanping injection.

### 3.3. Quality evaluation of included studies

**Figure 2.**
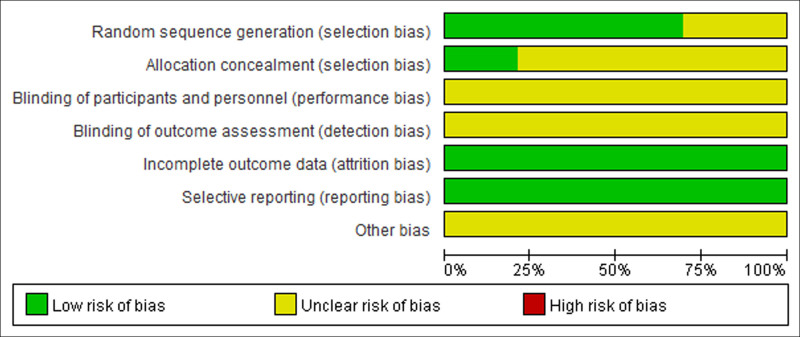
Percentages of items of included articles that produced risks of bias.

Among the 33 included studies,^[9–41]^ 24 studies^[9–14,16,18,19,21,24–32,35–37,39,41]^ were randomized. Specifically, 22 studies^[9–14,16,18,19,21,24–28,30,32,35–37,39,41]^ were randomized using a number table, and 2 studies^[29,31]^ were randomized by lottery. The risk of bias in this literatures were determined to be low risk. Blinding method of remaining studies was not reported, and the risk of bias was assessed to be uncertain. None of the included studies reported allocation concealment. There was no evidence of incomplete result data, and no selective reporting of outcome measures or other sources of bias. The literature quality assessment is presented in Figure 2.

### 3.4. Evidence network

**Figure 3.**
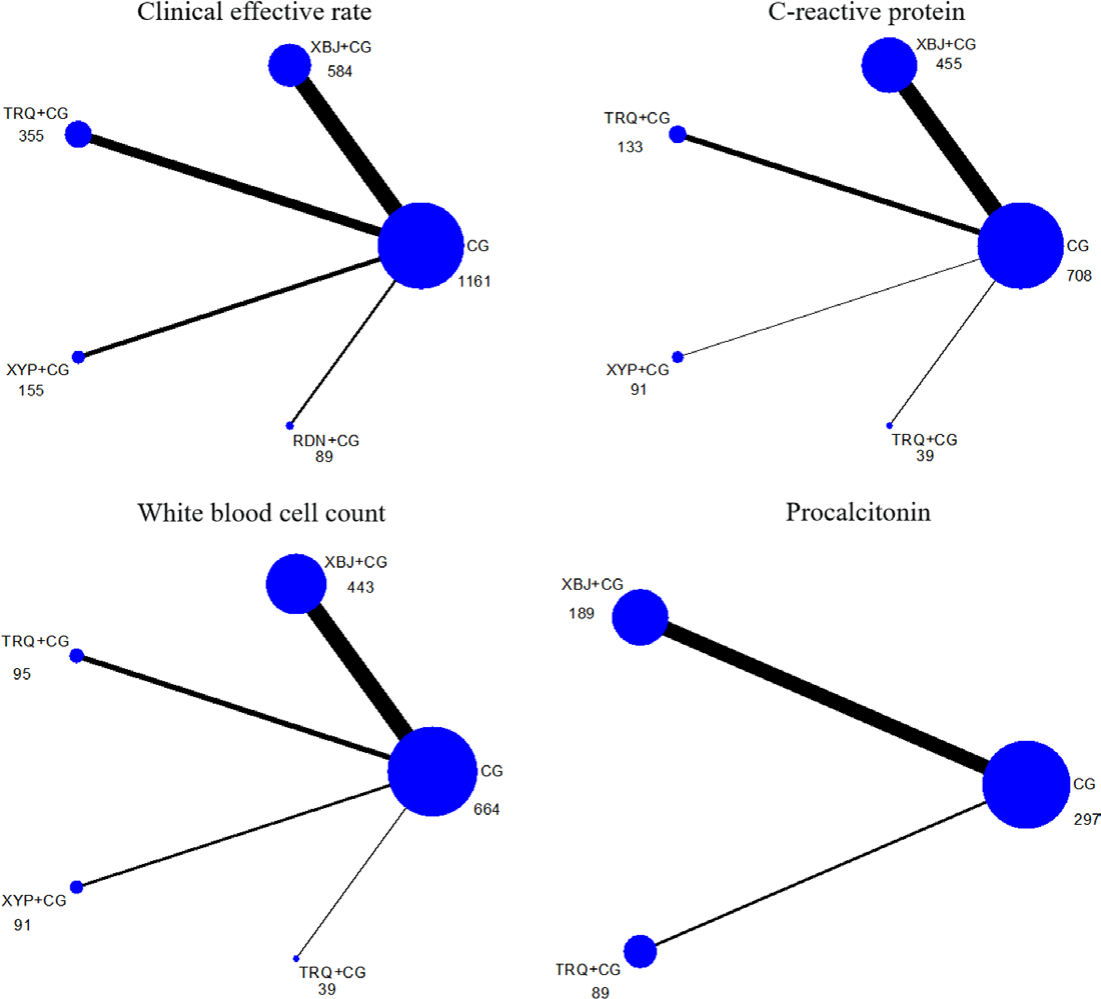
Evidence network of each outcome indicators.

The network relationship of the 4 Chinese medicine injections combined with conventional drugs treatment of senile severe pneumonia is shown in Figure 3. There is no closed ring. The consistency test results show that the consistency is good. The line segment thickness represents the included study of each intervention. The circular area represents the sample size using this measure.

### 3.5. Network meta-analysis

#### 3.5.1. Clinical effective rate

Thirty-one studies reported the clinical effective rate, involving 4 Chinese herbal injections. Network meta-analysis results showed that Xuebijing injection (OR = 2.66, 95% CI [1.61, 4.40]), Tanreqing injection (OR = 3.97, 95% CI [2.06, 7.64]), Xiyanping injection (OR = 3.9, 95% CI [1.47, 10.32]), and Reduning injection (OR = 5.37, 95% CI [1.24, 23.36]) combined with conventional treatment were superior to conventional treatment alone, and the differences were statistically significant (*P* < .05), in Table 2.

**Table 2:** Network meta-analysis of clinical effective rate.

Interventions	OR (95% CI)				
	CG	XBJ + CG	TRQ + CG	XYP + CG	RDN + CG
CG	–				
XBJ + CG	2.66 (1.61,4.40)^△^	–			
TRQ + CG	3.97 (2.06,7.64)^△^	0.67 (0.30,1.52)	–		
XYP + CG	3.90 (1.47,10.32)^△^	0.68 (0.23,2.03)	1.02 (0.32,3.26)	–	
RDN + CG	5.37 (1.24,23.36)^△^	0.50 (0.11,2.34)	0.74 (0.15,3.68)	0.73 (0.13,4.22)	–

The OR value and 95% CI of the comparison between the 2 interventions corresponding to the list and row of each data: when the OR value is >1, it indicates that the intervention in the column is inferior to the intervention in the row; when the OR value is <1, it indicates that the intervention in the column is superior to the intervention in the row; when the 95% CI does not include 1, it indicates that the difference is statistically significant, and it is indicated in the table “△.” The MD value and 95% CI of the comparison between the 2 interventions corresponding to the row and row of each data: where the MD value <0 indicates that the intervention in the column is inferior to the intervention in the row, the MD >0 indicates that the intervention in the column is superior to the intervention in the row, and the 95% CI excluding 0 indicates that the difference is statistically significant, and it is indicated in the table as “△”.

95% CI = 95% confidence interval, OR = odds ratio.

#### 3.5.2. White blood cell count

Sixteen studies reported WBC count values, involving 4 Chinese herbal injections. Network meta-analysis results showed that Xuebijing injection (MD = ‐1.89, 95% CI [‐2.95, ‐0.83]), Tanreqing injection (MD = ‐2.78, 95% CI [‐5.07, ‐0.50]), Xiyanping injection (MD = ‐2.9, 95% CI [‐5.04, ‐0.75]) combined with conventional treatment were superior to conventional treatment alone, and the difference was statistically significant (*P* < .05), only Reduning injection (MD = ‐1.87, 95% CI [‐5.47, 1.73]) was not statistically significant, which only included one literature, which may bias the results shown in Table 3.

**Table 3:** Network meta-analysis of white blood cell count.

Interventions	MD (95% CI)				
	CG	XBJ + CG	TRQ + CG	XYP + CG	RDN + CG
CG	0				
XBJ + CG	‐1.89 (‐2.95,‐0.83)^△^	0			
TRQ + CG	‐2.78 (‐5.07,‐0.50)^△^	0.90 (‐1.62,3.42)	0		
XYP + CG	‐2.90 (‐5.04,‐0.75)^△^	1.01 (‐1.38,3.40)	0.11 (‐3.02,3.25)	0	
RDN + CG	‐1.87 (‐5.47,1.73)	‐0.02 (‐3.77,3.73)	‐0.91 (‐5.18,3.35)	‐1.03 (‐5.21,3.16)	0

The OR value and 95% CI of the comparison between the 2 interventions corresponding to the list and row of each data: when the OR value is >1, it indicates that the intervention in the column is inferior to the intervention in the row; when the OR value is <1, it indicates that the intervention in the column is superior to the intervention in the row; when the 95% CI does not include 1, it indicates that the difference is statistically significant, and it is indicated in the table “△.” The MD value and 95% CI of the comparison between the 2 interventions corresponding to the row and row of each data: where the MD value <0 indicates that the intervention in the column is inferior to the intervention in the row, the MD >0 indicates that the intervention in the column is superior to the intervention in the row, and the 95% CI excluding 0 indicates that the difference is statistically significant, and it is indicated in the table as “△”.

95% CI = 95% confidence interval.

#### 3.5.3. C-reactive protein

Nineteen studies reported CRP, involving 4 Chinese herbal injections. Network meta-analysis results showed that only Xuebijing injection (MD = ‐10.19, 95% CI [‐14.28, ‐6.10]) and Xiyanping injection (MD = 20.02, 95% CI [‐32.55, ‐9.49]) combined with conventional treatment were superior to conventional treatment alone, and the differences were statistically significant (*P* < .05), and the others were not statistically significant. As shown in Table 4.

**Table 4:** Network meta-analysis of C-reactive protein.

Interventions	MD (95% CI)				
	CG	XBJ + CG	TRQ + CG	XYP + CG	RDN + CG
CG	0				
XBJ + CG	‐10.19 (‐14.28,‐6.10)^△^	0			
TRQ + CG	‐0.45 (‐3.58,2.68)	‐9.74 (‐14.88,‐4.60)^△^	0		
XYP + CG	‐21.02 (‐32.55,-9.49)^△^	10.83 (‐1.40,23.07)	20.57 (8.62,32.52)^△^	0	
RDN + CG	‐9.32 (‐26.17,7.53)	‐0.87 (‐18.21,16.47)	8.87 (‐8.27,26.01)	‐11.70 (‐32.12,8.72)	0

The OR value and 95% CI of the comparison between the 2 interventions corresponding to the list and row of each data: when the OR value is >1, it indicates that the intervention in the column is inferior to the intervention in the row; when the OR value is <1, it indicates that the intervention in the column is superior to the intervention in the row; when the 95% CI does not include 1, it indicates that the difference is statistically significant, and it is indicated in the table “△.” The MD value and 95% CI of the comparison between the 2 interventions corresponding to the row and row of each data: where the MD value <0 indicates that the intervention in the column is inferior to the intervention in the row, the MD >0 indicates that the intervention in the column is superior to the intervention in the row, and the 95% CI excluding 0 indicates that the difference is statistically significant, and it is indicated in the table as “△”.

95% CI = 95% confidence interval.

#### 3.5.4. Procalcitonin

PCT was reported in 7 included studies, involving 2 Chinese herbal injections. Network meta-analysis results showed that only Xuebijing injection (MD = ‐1.36, 95% CI [‐1.82, ‐0.89]) combined with conventional treatment was superior to conventional treatment alone, and the difference was statistically significant (*P* < .05), shown in Table 5.

**Table 5:** Network meta-analysis of procalcitonin.

Interventions	MD (95% CI)		
	CG	XBJ + CG	TRQ + CG
CG	0		
XBJ + CG	‐1.36 (‐1.82,‐0.89)△	0	
TRQ + CG	‐0.32 (‐0.69,0.05)	‐1.03 (‐1.59,‐0.48)△	0

The OR value and 95% CI of the comparison between the 2 interventions corresponding to the list and row of each data: when the OR value is >1, it indicates that the intervention in the column is inferior to the intervention in the row; when the OR value is <1, it indicates that the intervention in the column is superior to the intervention in the row; when the 95% CI does not include 1, it indicates that the difference is statistically significant, and it is indicated in the table “△.” The MD value and 95% CI of the comparison between the 2 interventions corresponding to the row and row of each data: where the MD value <0 indicates that the intervention in the column is inferior to the intervention in the row, the MD >0 indicates that the intervention in the column is superior to the intervention in the row, and the 95% CI excluding 0 indicates that the difference is statistically significant, in the table “△” indicates.

95% CI = 95% confidence interval, OR = odds ratio.

### 3.6. Order of network meta-analysis results

#### 3.6.1. Clinical effective rate

**Figure 4.**
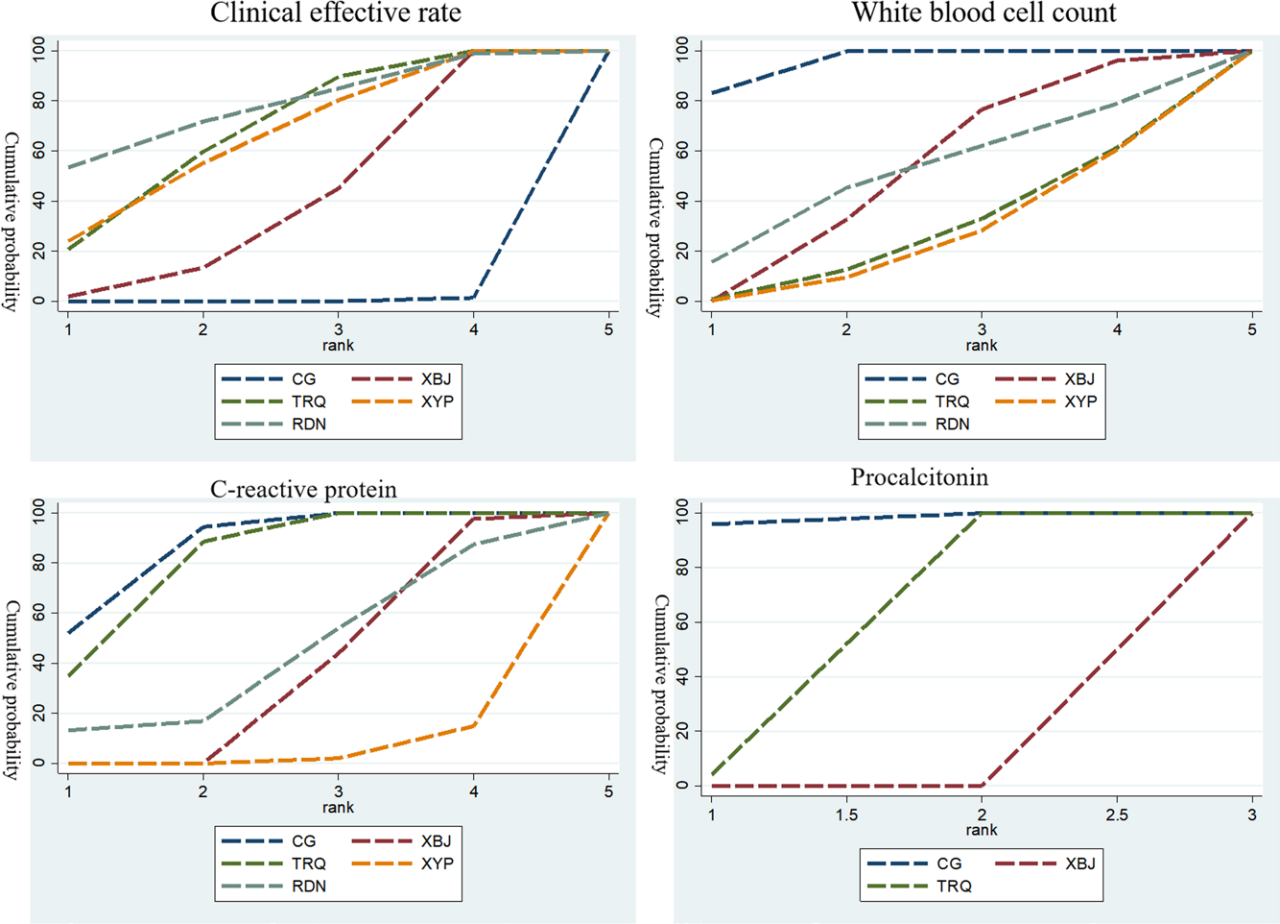
Cumulative probability rank.

The SUCRA ranking reveals that the combination of Reduning injection and conventional treatment is the best intervention measure in terms of clinical effectiveness rate: Reduning injection + conventional treatment (SUCRA = 77.3%) > Tanreqing injection + conventional treatment (SUCRA = 67.5%) > Xiyanping injection + conventional treatment (SUCRA = 64.8%) > Xuebijing injection + conventional treatment (SUCRA = 40.1%) > conventional treatment alone (SUCRA = 0.3%). As shown in Figure 4.

#### 3.6.2. White blood cell count

Since an increase in WBC count indicates a stronger inflammatory response. And what the SUCRA probability graph reflects is the trend of WBC increase. The higher the probability, the larger the WBC count value is compared to other values. However, it is the effect of the decrease in WBC count value that truly reflects the clinical efficacy. Therefore, in terms of reducing the WBC count value, the lower the SUCRA probability, the better the clinical efficacy. The SUCRA ranking shows that the combination of Xiyanping injection and conventional treatment is the best intervention measure in terms of clinical effectiveness rate: Xiyanping injection + conventional treatment (SUCRA = 24.9%) < Tanreqing injection + conventional treatment (SUCRA = 27.7%) < Reduning injection + conventional treatment (SUCRA = 50.3%) < Xuebijing injection + conventional treatment (SUCRA = 51.3%) < conventional treatment alone (SUCRA = 95.8%). As shown in Figure 4.

#### 3.6.3. C-reactive protein

Under normal circumstances, the concentration of CRP in serum is relatively low. However, when an inflammatory response takes place, it will trigger an elevation in the CRP content to modulate the body’s inflammatory reaction. As the inflammatory response intensifies, the level of this index changes. In the SUCRA probability graph, it reflects the degree of the inflammatory reaction. The higher the probability, the more severe the inflammatory reaction. Consequently, in terms of reducing the level of CRP, the lower the SUCRA probability, the more favorable the clinical outcome. The SUCRA ranking indicated that the combination of Xiyanping injection and conventional treatment was the optimal intervention for reducing CRP levels. Specifically, Xiyanping injection + conventional treatment (SUCRA = 4.2%) < Xuebijing injection + conventional treatment (SUCRA = 35.5%) < Reduning injection + conventional treatment (SUCRA = 42.9%) < Tanreqing injection + conventional treatment (SUCRA = 80.7%) < conventional treatment alone (SUCRA = 86.7%). As shown in Figure 4.

#### 3.6.4. Procalcitonin

In a manner analogous to the previous situation, it holds true that as the SUCRA probability ascends, the intensity of the inflammatory response amplifies. Consequently, when it comes to the level of PCT, a diminished SUCRA probability invariably heralds a more favorable clinical outcome. The SUCRA ranking demonstrated that the combination of Xuebijing injection and conventional treatment might be the most effective intervention for reducing PCT levels, with Xuebijing injection + conventional treatment (SUCRA = 0.0%) < Tanreqing injection + conventional treatment (SUCRA = 52.1%) < conventional treatment alone (SUCRA = 97.9%). As illustrated in Figure 4.

### 3.7. Publication bias test

**Figure 5.**
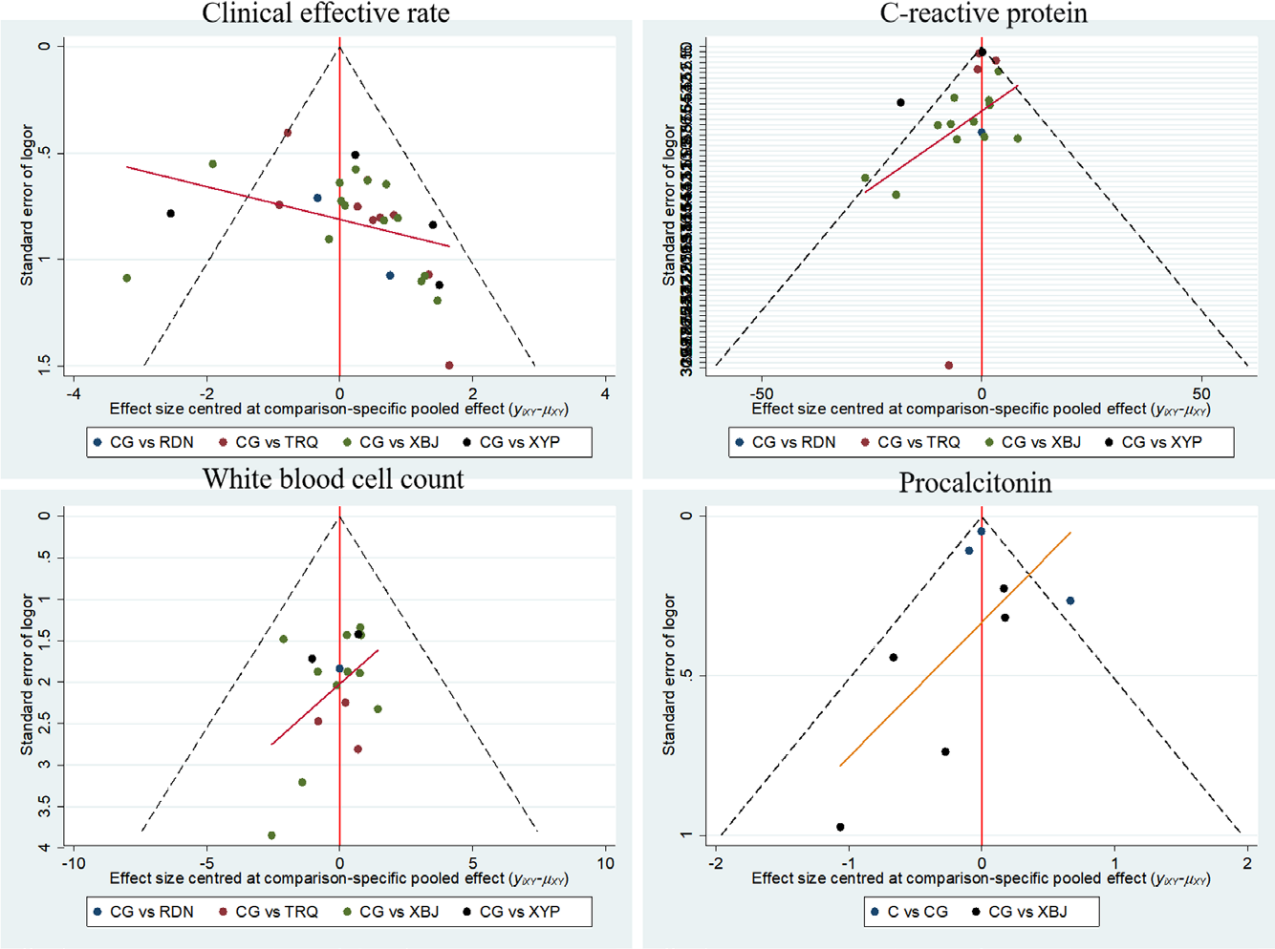
Funnel plot for correction comparison.

The funnel plot of “correction-comparison” was constructed for the 4 indices, namely the clinical response rate, WBC count, CRP, and PCT. The outcomes indicated that the symmetry was rather poor, implying that the incorporated studies might possess a certain degree of publication bias or a small sample effect. As depicted in Figure 5.

### 3.8. Safety

Out of the 33 studies that were included, 7 studies^[9,27,29,32,36,37,41]^ reported the incidence of adverse reactions. One study^[29]^ stated that there were no adverse reactions in the test group, and 2 studies^[32,41]^ reported no significant adverse reactions. As presented in Table 6.

**Table 6:** Occurrence of adverse reactions.

Included in the study	Intervention study	Control group	Experimental group
Xiao Qing et al^[9]^	XBJ + CG vs CG	2 patients had nausea 1 patient had a headache	1 patient had nausea 3 patients had itchy skin
Zhang Hongyan^[27]^	TRQ + CG vs CG	1 patient had nausea, 1 patient had a rash, and1 patient had diarrhea	1 had nausea, 1 rash and 1 vomiting
Xu Rugui^[29]^	TRQ + CG vs CG	Two cases had itchy skin	Not reported
Zhou Jing et al^[32]^	TRQ + CG vs CG	No significant adverse effects were seen	
Lai Daokin et al^[36]^	XYP + CG vs CG	3 patients had nausea, 3 had vomiting, 4 had diarrhea, and 5 had fever	2 patients had nausea, 2 vomiting, 6 diarrhea and 5 fever
Liu Rong et al.^[37]^	XYP + CG vs CG	7 patients had pungent cough, 2 had high blood pressure and 1 had hypoxemia	5 patients had irritant cough, 3 had elevated blood pressure and 1 had hypoxemia
Zhou Jing^[41]^	RDN + CG vs CG	No significant adverse effects were seen	

## 4. Inconsistency test

All 4 outcome measures were two-arm trials without any closed loops, an inconsistency test was not necessary. Therefore, the consistency model was employed for the network meta-analysis in this study.

## 5. Discussion

Severe pneumonia in the elderly is a serious disease problem.^[42]^ The pathogenesis of senile severe pneumonia is complex, which seriously affects the quality of life of the elderly. There are also many extrapulmonary complications in elderly patients with severe pneumonia, which is a major difficulty in clinical treatment.^[43,44]^ Previous researches have shown that traditional Chinese medicine injection combined with conventional drugs in the treatment of senile severe pneumonia achieved better effect.^[45–54]^

Pathogenic bacteria invade lung tissue and cause infection, which can activate the inflammatory mediators response, a large quantity of inflammatory mediators such as PCT, WBC, and CRP are massively released, accelerate lung injury, lead to hypoxemia, and further cause systemic multi-organ function damage.^[55]^ In this study, 3 inflammatory indicators were used to evaluate the degree of reduction in the inflammatory response. Clinical response rate is used to evaluate overall efficacy. The results of network meta-analysis showed that TCM injection combined with conventional drugs more efficacious than conventional drugs in the treatment of senile severe pneumonia.

Reduning Injection combined with conventional drugs was the best intervention in terms of clinical effectiveness. Previous studies have found that Reduning injection was mainly used in clinical treatment of cough, high fever, and other upper respiratory tract infections caused by external wind and heat.^[56]^ Reduning injection had good anti-inflammatory effect. Some studies had found that Reduning injection could promote the differentiation of T-lymphocyte cells and enhance the immune function of the body.^[57]^ Respiratory dyspnea, cough and other symptoms of patients with respiratory syncytial virus pneumonia were significantly improved by Reduning injection. It also reduces serum levels of PCT, CRP, and the production of inflammatory cytokines.^[58,59]^

Xiyanping injection combined with conventional drugs may be the best intervention measure to reduce WBC count and CRP index. Research has revealed that Xiyanping injection is a patented traditional Chinese medicine with andrographolide sulfonate as its principal constituent.^[60]^ In clinical therapeutics, it exhibits bactericidal and antibacterial activities against *Diplococcus pneumoniae*, *Staphylococcus aureus*, and Shigella, while also possessing multiple effects including antipyretic, anti-inflammatory, antitussive, antiasthmatic, and the capacity to enhance the body’s immune function, conferring significant advantages in the treatment of respiratory disorders; andrographolide demonstrates a broad spectrum of biological activities, such as anti-infection, antiviral, antibacterial, and immunomodulatory functions. Studies have demonstrated that Xiyanping injection can suppress the inflammatory response by impeding the secretion of inflammatory cytokines and can diminish the levels of TNF-α, IL-6, IL-17A, and other proinflammatory cytokines.^[61,62]^

Xuebijing injection when combined with conventional treatment is likely to constitute the most optimal intervention for reducing PCT levels. Certain studies have ascertained^[63,64]^ that the active ingredients within Xuebijing injection comprise safflower flavin A, ferulic acid, danshensu, paeoniflorin, protocatechuic aldehyde, and ligustrazine, which possess the capabilities of diminishing endotoxin levels, suppressing the inflammatory response, reducing IL-6 and TNF-α, and modulating the immune response so as to govern the occurrence and development of infection.

Regarding adverse effects, studies have demonstrated that the adverse reactions of these 4 injections predominantly center around nausea, headache, vomiting, diarrhea, fever, palpitation, rash, and finger trembling. Nevertheless, the frequency of adverse events was lower than that in the conventional treatment group.

## 6. Limitations

This study is subject to several limitations. Firstly, only 3 articles were included, which is a rather limited number. This has, to some extent, undermined the reliability of the results. The sample size was 2598, which is relatively small and may give rise to bias. There were few secondary outcome measures. Moreover, it should be noted that the cell count may not increase even when the disease worsens, which might lead to “false negative” outcomes in the assessment of disease progression.^[59]^ The included studies were based on indirect comparisons, lacking direct comparison evidence among different traditional Chinese medicine injections. Consequently, the results might deviate from actual clinical medication scenarios, thereby reducing the reliability of the findings. Due to the individualized and diversified nature of traditional Chinese medicine therapy, this study combined the analysis of different types of traditional Chinese medicine, which could potentially impact the reliability of the meta-analysis results.

## 7. Conclusion and outlook

In conclusion, the combined application of traditional Chinese medicine injections on the basis of conventional Western medicine treatment is capable of enhancing the comprehensive clinical efficacy in the management of severe pneumonia among the elderly. Among these, Reduning injection exhibits certain advantages in terms of improving the cumulative ranking outcomes of the clinical effective rate. Xiyanping injection is mainly focused on reducing the levels of CRP and WBC. Meanwhile, Xuebijing injection is centered on reducing PCT. The findings of this study offer a reference for the utilization of clinical traditional Chinese medicine injections in the treatment of severe pneumonia among the elderly. However, it still has certain limitations. The ranking of efficacy does not comprehensively elucidate the pros and cons of clinical efficacy. Consequently, the conclusions of this study remain to be further verified through more large-sample, multicenter, and high-level randomized controlled trials in the future.

## Author contributions

**Methodology:** Jie Liu.

**Supervision:** Jie Liu.

**Validation:** Jie Liu.

**Writing – original draft:** Yangli Tu, Changbing Wu, Liang Chen, Yinzhi Cen.

**Writing – review & editing:** Yangli Tu, Changbing Wu, Jie Liu.

## References

[1] Zhang M Xiong P. Clinical effect of Xuanbai Chengqi decoction in the treatment of senile severe pneumonia with phlegm-heating and stagnation of lung type and its influence on inflammatory factors. Clin Res Pract. 2020;5:135–8.

[2] Li X Xing J Li J. Efficacy and value of serological markers in diagnosis and evaluation of community-acquired pneumonia in severely ill elderly. Mod J Integr Tradit Chin West Med. 2020;29:3039–42.

[3] Guo LL. Gram-negative bacilli infection and drug resistance in elderly patients with pneumonia in the medical intensive care unit. J China Med Univ. 2020;000906:1–28.

[4] Luo AH Liu Y. The effect of low-molecular-weight heparin combined with amikacin on the coagulation function and bacterial clearance in the treatment of patients with severe senile pneumonia. Pak J Med Sci. 2023;39:172–6. 36694771 10.12669/pjms.39.1.6627 PMC9842970

[5] Zhu CX. Clinical effect evaluation of moxifloxacin combined with cefoperazone sodium and sulbactam sodium in the treatment of severe pneumonia in the elderly. Chin Prac Med. 2024;19:102–5.

[6] Zhang H Li YM Zhang SP Wu J. Effect of ceftriaxone combined with glucocorticoids on ciliary clearance function and microinflammatory factors in elderly patients with severe pneumonia. Chin J Drug Appl Monit. 2024;21:514–8.

[7] Su H Liao CJ Jiang Y. Research progress on prevention and treatment of senile severe pneumonia by combining traditional Chinese and Western medicine. Inner Mongolia J Tradit Chin Med. 2022;41:159–62.

[8] Peng Z Jingjing Y. Pharmaceutical monitoring of 3 kinds of heat-clearing TCM injections. J Pharmacovigilance. 2022;19:441–5.

[9] Xiao Q Yu D. Evaluation of the clinical effect of Hembijing injection combined with meropenem in senile severe pneumonia and its effect on serum inflammatory factors. Chin J Ration Drug Use. 2020;17:47–51.

[10] Xiao HW Peng Y Zhou HW. Efficacy of Hembijing injection combined with piperacillin sodium sulbactam in treating severe pneumonia in the elderly and its effect on inflammatory factors and immune function. Chin J Gerontol. 2021;41:4218–21.

[11] Xu JJ Wang Y. Effect of Hembijing combined with linezolid in geriatric severe pneumonia and its effect on serum inflammatory factor levels in patients. Clin Med Eng. 2022;29:19–20.

[12] Yang HW Shi F Yu QP. Effect of Hembijing on serum STREM-1 level and CPIS score in elderly patients with severe pneumonia. Jiangxi Me. 2020;55:1008–11.

[13] Li XJ Zhou L. Effect of Hembijing combined with linezolid injection on serum levels of pulmonary surface active protein, matrix metalloproteinase and their tissue inhibitors in elderly patients with severe pneumonia. Prog Mod Biomed. 2018;18:4773–7.

[14] He W Li RY. Effect of integrated Chinese and Western medicine on clinical efficacy and serological indicators in elderly patients with severe pneumonia. Western J Tradit Chin Med. 2020;33:95–8.

[15] Guo LM. Efficacy of Hembijing injection in the treatment of senile severe pneumonia and its effect on the intestinal mucosal barrier and oxygen supply status in patients. Shaanxi J Tradit Chin Med. 2019;40:700–4.

[16] Chen JZ. Study on the efficacy of Hembijing injection combined with meropenem in elderly patients with severe pneumoni. Pract Geriatr. 2019;33:182–5.

[17] Zhuang L. Observation on the therapeutic effect of Hemobijing injection in the treatment of elderly patients with severe pneumonia. China Health Care Nutr. 2016;26:306.

[18] Zhu YJ. Clinical study of Hembijing injection for severe pneumonia in the elderly. Hebei Med. 2015;21:1683–5.

[19] Zhang X Wang SA. Efficacy and safety of Hembijing in severe pneumonia. Chin J Gerontol. 2015;35:1785–6.

[20] Xiao Y. Clinical effect of Hembijing in adjuvant treatment of severe pneumonia in the elderly. J Clin Psychosom Dis. 2015;10:246–7.

[21] Lv S. Hembijing treated 45 cases of severe pneumonia in the elderly and its effect on immune indicators. China Pharm. 2015;24:116–8.

[22] Liu JL. Clinical observation of 32 cases of senile severe pneumonia. World J Integr Tradit Chin West Med. 2013;8:1249–51.

[23] Xiao HY Xia WH. Efficacy observation of Xuebijing injection in elderly patients with severe pneumonia. China Health Nutr. 2013;7:490–1.

[24] Wang ZG Long YZ. Observation of the efficacy of Hembijing in the treatment of severe pneumonia in the elderly. Jiangxi Med. 2013;48:41–2.

[25] Shen MY Zhang HX. Clinical efficacy and safety observation of Xuebijing injection in elderly patients with severe pneumonia. China Med Industry Guide. 2012;14:45–6.

[26] Liu H. Evaluation of the efficacy of Xuebijing injection in elderly patients with severe pneumonia. Heilongjiang Med Sci. 2022;45:164–5.

[27] Zhang HY. Clinical study of sputum Reqing injection combined with piperacillin sulbactam for the treatment of severe pneumonia in the elderly. Study Integr Tradit Chin West Med. 2021;13:5–7.

[28] Liu XL; Member of Qin Qing. Clinical efficacy of sputum Reqing injection combined with piperacillin sulbactam in senile severe pneumonia and its effect on serum inflammatory factors. Int Med Health Guide. 2019;25:603–6.

[29] Xu RG. The clinical effect of sputum Reqing injection in treating severe pneumonia. Famous Doctors. 2018;37:37.

[30] Chen CM. Clinical study of sputum Reqing injection assisted antibiotic step descending treatment of severe pneumonia in the elderly. Health Must Read. 2018;15:71.

[31] Li G Wang SF. Clinical study of sputum Reqing injection assisted antibiotic step descending treatment of severe pneumonia in the elderly. Health Care Nutr China. 2018;28:41.

[32] Zhou J Shi ZD. Clinical efficacy of sputum Reqing injection for senile severe pneumonia and its effect on serum hypersensitivity C-reactive protein and procalcitonin. Laborat Med Clin Med. 2017;14:1637–9.

[33] Gu Q. Study on the clinical effect of sputum Reqing injection in treating severe pneumonia. Shenzhen J Integr Tradit Chin West Med. 2015;25:17–8.

[34] Zhang P Chen JH. Observation of the efficacy of sputum Reqing injection in treating senile severe pneumonia. Everybody Health (Mid-Term Ed). 2014;4:188.

[35] Wu XD. Clinical observation of sputum Reqing injection assisted with antibiotic step-down therapy for severe pneumonia in the elderly. J Clin Pulm Sci. 2012;17:1981–2.

[36] Lai DJ He FY. Clinical efficacy and safety of taazobactam sodium with piperacillin tam sodium combined with Xi Yan Ping in severe pneumonia in elderly. Chin J Clin Rational Drug Use. 2020;13:66–8.

[37] Liu R Li J. Clinical observation of severe pneumonia. J Emerg Tradit Chin. 2019;28:109–11.

[38] Hou YW. Clinical analysis of 62 cases of elderly severe pneumonia. Everybody Health (Mid-Term Ed). 2016;10:164.

[39] Zhang LL Wang GL. Clinical observation of Xiyanping injection for senile severe pneumonia. J Emerg Tradit Chin. 2015;24:2289–90.

[40] Ding F. Effect of thermoNing on lung function and immune function in elderly patients with severe pneumonia. China Pract Med J. 2017;44:119–22.

[41] Zhou J. Clinical observation of Revenom injection for senile severe pneumonia. Chin J Pract Med. 2017;28:1013–4.

[42] Li YF Zhang SJ. Analysis of the clinical characteristics and predictive indicators of severe pneumonia in the elderly population. Geriatr Med Health Care. 2022;28:589–93.

[43] Pan LP Liu ZB Wu M. Effect of lncRNA MALAT1 expression on survival status of elderly patients with severe pneumonia. Eur Rev Med Pharmacol Sci. 2020;24:3959–64. 32329872 10.26355/eurrev_202004_20865

[44] Liu DX Jiang Q. Distribution and risk factors of ventilator-associated pneumonia in elderly ICU patients. Pract Prev Med. 2022;29:1381–4.

[45] Cao CG Hen ZL Kuang SN. Reduning injection combined with western medicine for pneumonia: a protocol for systematic review and meta-analysis. Medicine (Baltim). 2020;99:22757. 10.1097/MD.0000000000022757 PMC7581155 33120780

[46] Luo W Liu Y Zhang Q Zhong H Deng J. Effect of traditional Chinese medicine injections on severe pneumonia: a protocol for systematic review and meta-analysis. Medicine (Baltim). 2020;99:e22012. 10.1097/MD.0000000000022012 PMC7523816 32991404

[47] Shi SH Wang F Chen BN. Efficacy and safety of Shenfu injection for severe pneumonia in the elderly: a systematic review and meta-analysis based on western and eastern medicine. Front Pharmacol. 2022;13:779942. 36091817 10.3389/fphar.2022.779942 PMC9454296

[48] Song YL Yao C Yao YM. Xuebijing injection versus placebo for critically ill patients with severe community-acquired pneumonia: a randomized controlled trial. Crit Care Med. 2019;47:735–43. 31162191 10.1097/CCM.0000000000003842 PMC6727951

[49] Wang L Fan YH Xu JY. The efficacy and safety of Tanreqing injection combined with western medicine for severe pneumonia: a protocol for systematic review and meta-analysis. Medicine (Baltim). 2020;99:22010. 10.1097/MD.0000000000022010 PMC7458256 32871955

[50] Zhang YK Lu P Qin H. Traditional Chinese medicine combined with pulmonary drug delivery system and idiopathic pulmonary fibrosis: rationale and therapeutic potential. Biomed Pharmacother. 2021;133:111072. 33378971 10.1016/j.biopha.2020.111072 PMC7836923

[51] Dong YB Zhang XL Mao LN. Meta-analysis of the efficacy of high-dose ambroxol combined with fibrobronchoscopic lavage and aspiration for treating severe geriatric pneumonia. Jiangsu Pharm. 2019;45:444–9.

[52] Lu P Zhu Y. Meta-analysis of the efficacy of ambroxol hydrochloride combined with sputum monitor and high-dose ambroxol hydrochloride combined with bronchoscopy in geriatric severe pneumonia. China J Endosc. 2020;26:61–73.

[53] Xu YQ Jiang ZJ Huang WD. Systematic evaluation of the efficacy and safety of four drugs for the adjuvant treatment of severe pneumonia in the elderly – a network-based meta-analysis. Chin J Disaster Med. 2021;9:745–50.

[54] Zhang K Zhang YQ Xie K. Meta-analysis of a randomized controlled study of Hemobijing injection for severe pneumonia in the elder. Tradit Chin Drug Res Clin Pharmacol. 2020;31:483–9.

[55] Yana GS Tsubouchi H Miura A. The impacts of cellular senescence in elderly pneumonia and in age-related lung diseases that increase the risk of respiratory infections. Int J Mol Sci . 2017;18:1–16. 10.3390/ijms18030503 PMC5372519 28245616

[56] Li CM Wang CC Fan YX. Molecular epidemiology of KPC-2 K. pneumoniae produced by intestinal colonization in critically ill elderly patients. Chin J Nosocomiol. 2022;32:2401–5.

[57] Liu N Zhong Q Dong HJ. Effect of medication guidance based on CPIS on medication time and DDDs in elderly patients with severe pneumonia caused by bacterial infection. Chin J Gerontol. 2022;42:3187–9.

[58] Ye XL Tang CC Liu H. Thertoin injection protected influenza mice by inhibiting inflammatory cell infiltration in the lungs and reducing cytokine storm. J Chin Mater Med. 2022;47:4698–706. 10.19540/j.cnki.cjcmm.20220427.402 36164877

[59] Li YT Zhou DY. Clinical efficacy of thermoNing injection in severe pneumonia with sepsis. Clinical J Chin Med. 2022;14:89–91.

[60] Liu TF Du N Zhang YF. Progress in the synthesis and activity of andrographolide derivatives. Nat Prod Res Dev. 2022;7:1–29.

[61] Wang ZF Zhang HC Xie YH. Expert consensus on the clinical application of Xiyanping injection in the treatment of respiratory infectious diseases (adult version). J Chin Mater Med. 2019;44:5282–6. 10.19540/j.cnki.cjcmm.20191105.502 32237369

[62] Ni SJ. Factors influencing the prognosis of severe pneumonia in the elderly, the diagnostic value of serum NLR and its correlation with the degree of disease. Med Innov China. 2022;19:106–11.

[63] Zhou HG Zhu H. Clinical study of Xuebijing injection for the treatment of senile severe pneumonia combined with heart failure. Asia Pacific Tradit Med. 2022;18:92–5.

[64] Ding WC Juan C Haoyu L. Based on network pharmacology and in vitro experiments to investigate the mechanism of Hembijing injection in the treatment of sepsis-related ARDS. J Chin Mater Med. 2023;48:3345–59. 10.19540/j.cnki.cjcmm.20230202.703 37382018

